# Maternal Care Behavior and Its Consequences in Competition

**DOI:** 10.3390/insects15040236

**Published:** 2024-03-29

**Authors:** Guang-Yun Li, Yu-Chuang Li, Huai Liu

**Affiliations:** 1 Key Laboratory of Entomology and Pest Control Engineering, College of Plant Protection, Southwest University, Chongqing 400715, China; 2 Key Laboratory of Agricultural Biosafety and Green Production of Upper Yangtze River (Ministry of Education), Southwest University, Chongqing 400715, China

**Keywords:** predatory mites, maternal care, competitor, survival, biological control

## Abstract

**Simple Summary:**

The maternal care behavior of the predatory mite *Cheyletus eruditus* (Schrank) and its impact on offspring survival and competition were investigated. The findings revealed that females exhibited egg-guarding behaviors, with increased maternal care efforts when interspecific competitors were present. The study demonstrated that egg masses were more vulnerable to predation in the absence of females, highlighting the importance of maternal care behaviors for offspring survival. Furthermore, the presence of guarding females increased egg survival rates and negatively impacted the survival of both conspecific and heterospecific competitors, resulting in higher mortality rates. These results emphasize the ecological significance of maternal care behaviors of *C. eruditus* and provide valuable insights for pest management with predatory mites.

**Abstract:**

Parental care behavior has evolved as a life history strategy to improve reproductive success, particularly in organisms facing challenging environments. However, the variation in maternal care, such as egg-guarding behavior in response to the social environment and the associated ecological consequence of competition, remains largely unknown. This study addresses a gap in current knowledge by examining the plasticity of maternal care behavior in the predatory mite *C. eruditus* and its impact on offspring survival and intra- and interspecific competition. Our results demonstrated that the reproductive females frequently exhibit egg-guarding behaviors, with enhanced maternal care efforts when the interspecific competitor is present. Egg masses are significantly more vulnerable to predation in the absence of maternal care. Guarding females increased egg survival rates and adversely influenced the survival of both con- and heterospecific competitors, with higher mortality rates being detected. Our findings highlight the ecological significance of maternal care behaviors and suggest that releasing *C. eruditus* and *Neoseiulus cucumeris* (Oudemans) together is not recommended for pest management in storage products.

## 1. Introduction 

Animals in nature face constant challenges such as predation pressure, harsh environments, or fluctuating food availability [1,2]. To enhance fitness, many species exhibit parental care behaviors to increase the survival rate of their offspring, thus increasing their own reproductive success [3]. These behaviors range from simple acts, like guarding the nest, to more complex ones, such as feeding and teaching the young [3,4,5,6,7,8]. Although these behaviors are often costly in terms of time and energy for the parents, they are critical for offspring survival, especially in environments where their chances of survival are extremely low without parental protection [9,10]. 

Cannibalism and intraguild predation, as unique forms of predation, are common in many species and have been proven to threaten the survival of animals, particularly the earliest stage in a population, such as eggs. Egg cannibalism was proposed as the selective force behind parental care in thrips *Elaphrothrips tuberculatus* (Hood) [11] and milkweed leaf beetle *Labidomera clivicollis* (Kirby) [12], influencing parental investment. Egg guarding is a prevalent strategy employed to protect offspring from conspecific predation. This behavior has been reported across many taxa, including fish, reptiles, amphibians, birds, and invertebrates [8,13,14,15,16,17]. The hypothesis is that this behavior has evolved to mitigate the risk of cannibalism. Much research has clarified how egg-guarding behavior influences the survival of offspring. An intriguing example is the flagfish *Jordanella floridae* (Goode and Bean), a species that has recently evolved paternal care. Male nest guarding increased egg survivorship when females and predatory Gambusia were present [13]. However, when males were alone with eggs, egg survivorship was low, indicating that males cannibalized eggs regardless of whether these eggs were healthy or diseased. This finding underscores that parental behavior strategies are significantly context-dependent. However, the plasticity of egg-guarding behavior under various circumstances remains unexplored despite numerous studies examining the benefits and costs of egg-guarding behavior for offspring and parents in many animals [4,13,18,19,20,21]. Hence, it would be important to investigate flexible egg-guarding behavior in animals to fully understand the net effects of egg-guarding and its related ecological consequences.

*Cheyletus eruditus* is a prostigmatid predator known for its nest behavior [22]. Nesting females remained in the nest, staying on or close to their egg mass [23,24]. When removed from their nest, they attempted to return. Once they perceived a potential competitor or intruders, the nesting female displayed attacking behavior, including forward body thrust and a strong clamping motion of the pincer-like pedipalps [22]. Despite the fact that the females make great efforts to prevent their eggs from predation, cannibalism was witnessed. The mothers even consume their eggs and larvae when they encounter a shortage of food [25,26]. In addition to cannibalistic conspecifics, they might also face intraguild predation from other co-existing predators. Although nesting behavior has been noted, how parental care behavior and the presence of competitors shape the survival of eggs remains to be fully understood. 

Since *C. eruditus* was identified as a predator of acarid mites, it has been commercially mass-produced and employed as a biological control agent in practice [27,28,29]. Extensive research has documented its efficiency not only as a predator of storage mites from the family Acaridae and Glycyphagidae [27,30,31,32,33] but also as a promising candidate for controlling the poultry red mite *Dermanyssus gallinae* (De Geer) [28,34]. To fully understand its potential as a biological control agent, an in-depth understanding of the life history strategies, such as egg-guarding behavior, is urgently needed. 

In this study, we initially investigated the prevalence of egg-guarding behavior among females to further elucidate the phenomenon. Based on this, a full factorial design incorporating both the presence of maternal care and the type of predator (i.e., conspecific and heterospecific competitor) was employed to assess how egg-guarding behavior varies within a social environment. We expected that the intensity of egg-guarding behavior would be higher for female *C. eruditus* together with heterospecific competitors. We further determined the proportion of egg masses remaining intact to clarify how maternal care and the presence of competitors influence egg survival. We hypothesize that maternal care will effectively safeguard their eggs from predation by competitors. We also examined whether egg-guarding females attacked competitors and how this behavior influences competitor survival. Given that cannibalism and intraguild predation in this species is prevailing, the death of competitors would be observed in treatments with maternal care. 

## 2. Materials and Methods

### 2.1. Mites Colony 

*Cheyletus eruditus* was collected from the contaminated *Neoseiulus cucumeris* (Oudemans) population, and a colony was established on *Tyrophagus putrescentiae* (Schrank) with bran and the dry yeast *Saccharomyces cerevisiae* (Meyen ex EC Hansen) in a food box (750 mL). The box was placed on a black plastic sheet kept on a sponge half-submerged in salt water to prevent the predatory mite from escaping. 

*Neoseiulus cucumeris* was commercially obtained from Fujian Yanxuan Biocontrol Technology Co., Ltd. (Minhou County, China), and its population was maintained in the laboratory by regularly replenishing new wheat bran and yeast. The population was mass reared with the same method as *C. eruditus*. 

The colony of *T. putrescentiae* was maintained in a Petri dish (9 cm in diameter) with yeast. The population was also isolated by salty water. All these three populations were maintained in a climate chamber at 25 ± 1 °C, 80 ± 10% RH, and 16L:8D photoperiods. 

### 2.2. Experimental Cells

The experimental cell consists of a transparent acrylic square plate (30 mm × 30 mm × 3 mm) with a circular hole (16 mm in diameter) in the middle, which was sealed on the bottom by a piece of metal mesh (50 microns) to allow ventilation and on the top with a piece of transparent glass to facilitate observation. They were fixed together by a pair of long-tailed metal clips.

### 2.3. Maternal Behavior Observation 

**Figure 1:**
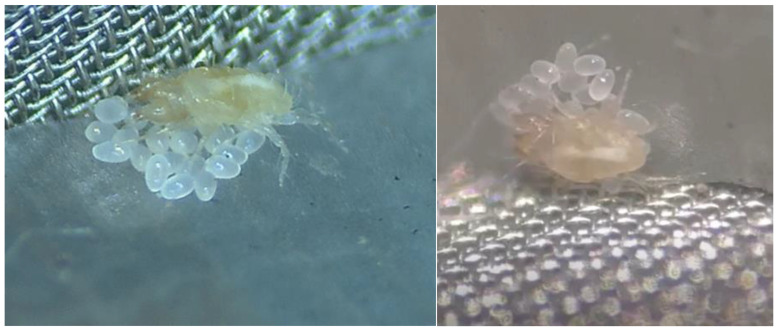
Parental care behavior of the predatory mite *Cheyletus eruditus*.

**Figure 2:**
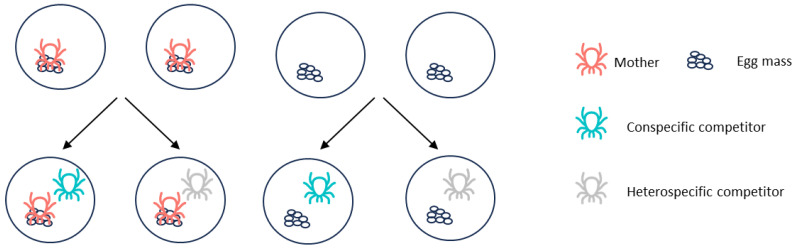
Diagram of the design of experiment 2.

To obtain female predatory mites of *C. eruditus* in their reproductive stage, females from the laboratory population were collected. They were kept one in each experimental cell, fed ample prey, and allowed to lay eggs for a few days. Then, females with egg masses were used in the following experiment. The maternal behavior of females in each cell was checked and classified into three groups: (1) Egg guarding behavior: the females stayed on the egg mass or with their body in contact with their eggs (Figure 1); (2) egg attendance: the female was near their egg mass within the distance of their body length; (3) not caring: the females were resting far away from their egg mass. The maternal behavior of 185 females was observed. 

### 2.4. Influence of Maternal Care on Offspring and Competitors

The experimental cells with females showing maternal care behavior were used in the following experiment. Before the experiment, each cell was checked with one female and seven eggs being left, while surplus eggs were removed without disturbing their mothers. Ample prey was added in each cell to avoid cannibalism of the mother to their eggs. Then, they were randomly assigned into two groups evenly. In one group, the mothers of eggs were removed, resulting in cells without maternal care. In the other group, the eggs were kept together with their mother. Then, each cell in these two groups was introduced to a conspecific female predatory mite *N. cucumeris*, or a female heterospecific predator, generating four treatments in total (Figure 2): (i) cells without maternal care but having conspecific predator, (ii) cells without maternal care but having heterospecific predators, (iii) cells with maternal care and conspecific predators, (iv) cells with maternal care and heterospecific predators. There were 36, 31, 36, and 38 replicates, respectively. Additionally, to distinguish the mother from its conspecific predator, the latter was carefully marked with green color by a 000 brush. Meanwhile, the gravid females of the heterospecific competitor *N. cucumeris* were employed in this experiment, and the cells were checked 24 h after their introduction. Firstly, whether the egg masses of *C. eruditus* had been attacked were checked for each cell, regardless of the number of eggs consumed. Secondly, we checked the survival of the introduced competitor to see whether cannibalism or intraguild predation occurred. Thirdly, we also recorded whether eggs of the heterospecific competitor *N. cucumeris* were found in the cells. 

### 2.5. Data Analysis 

The proportion of mothers demonstrating different reproductive behaviors, including egg attendance, egg guarding, and others, were compared with the Chi-Square Test to clarify the difference with the function “chisq.test”. The number of *N. cucumeris* produced eggs during the experiment in the presence and absence of *C. eruditus* were also compared with the Chi-Square Test. To determine the influence of maternal care and the type of competitor (conspecific and heterospecific), a General linear model with a link function binomial was conducted to find whether the egg mass was attacked and whether the competitor survived. The proportion of cells in which the *C. eruditus* eggs remained intact or the competitors were still alive, respectively, were subjected to the Fisher’s Exact Test R base function “fisher.test”. To clarify the difference across treatments, the post hoc tests of homogeneity were performed with the function “pairwise_fisher_test”. Significance levels were set at the 5% level. All the data analyses were conducted with R (version 4.0.1) [35]. 

## 3. Results

**Figure 3:**
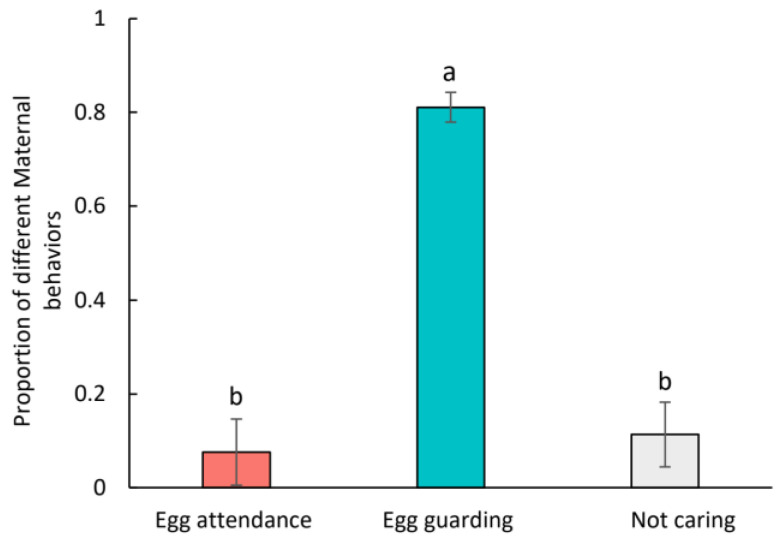
The proportion of female predatory mites *Cheyletus eruditus* demonstrating different behaviors during their reproductive period. Different lowercase letters indicate significant difference among treatments at the 5% level.

There was a significant variation in the number of females exhibiting different behaviors. Most of them demonstrated egg-guarding behavior, making up 81.08% of the total number of females checked, significantly higher than the mites showing other behaviors (χ^2^ = 190.19, *p* < 0.001; Figure 3). About 7.57% of the females exhibited egg attendance behavior, while the remaining 11.35% were observed far from their egg mass. The data of the latter two behaviors did not differ significantly between them (χ^2^ = 1.1360, *p* = 0.2864; Figure 3). 

**Figure 4:**
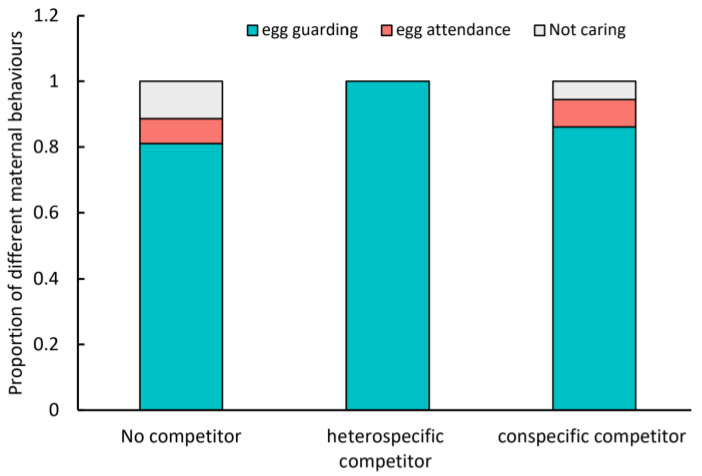
Influences of competitors on the maternal care behaviors of *Cheyletus eruditus* towards eggs.

The degree of maternal care was influenced by competitors, but the influence was only marginally significant (χ^2^ = 9.3911, *p* = 0.05203; Figure 4), with mothers caged together with heterospecific competitor all showed egg guarding compared with that exposed to no competitors (χ^2^ = 8.5276, *p* = 0.014). While the treatment without competitors did not have obvious differences with that caged together with conspecific competitors (*p* > 0.05).

**Figure 5:**
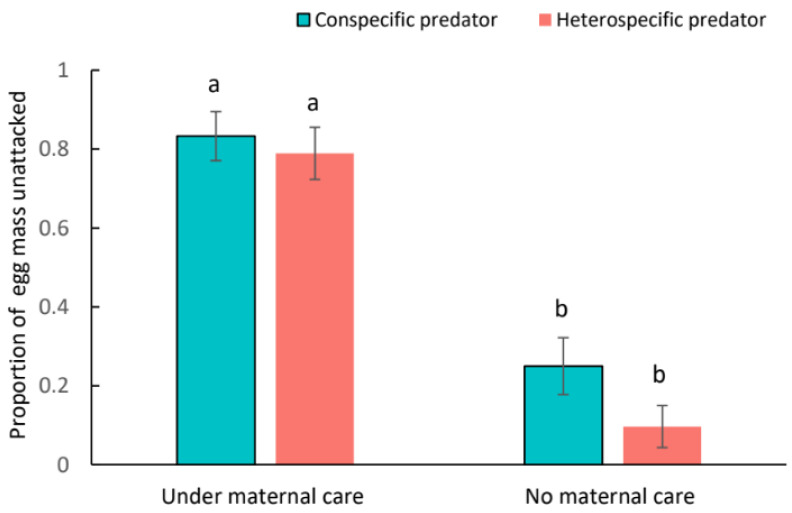
The influences of maternal care behavior on the survival of *Cheyletus eruditus* offspring in the presence of conspecific and heterospecific competitors. Different lowercase letters indicate significant difference among treatments at the 5% level.

The presence of maternal care showed an obvious influence on whether the *C. eruditus* egg masses were attacked. With the presence of their mothers, the probability of eggs being attacked was significantly lower than that exposed to competitors (*Z* = 4.590, *p* < 0.001; Figure 5). Among all these treatments, the proportion of egg masses intact was significantly higher in treatments under maternal care than those without, irrespective of the type of competitors (*p* < 0.001; Figure 5). However, there was no significant difference between the proportion of egg masses not attacked by conspecific and heterospecific competitors (*Z* = 1.578, *p* = 0.11449). Also, the interaction effects between maternal care and the type of competitors on the probability of egg mass being attacked were insignificant (*Z* = −0.906, *p* = 0.36516). 

**Figure 6:**
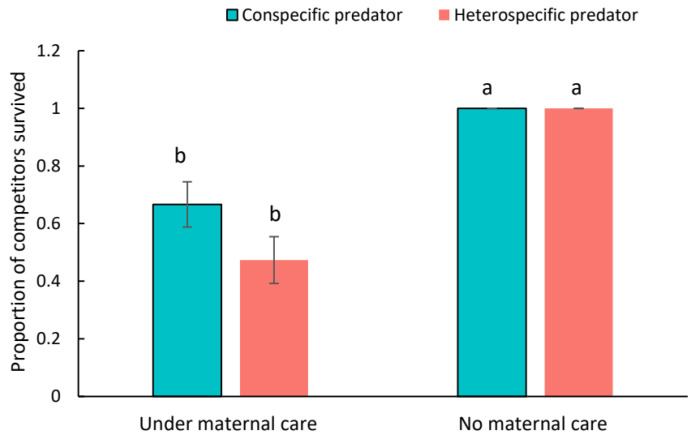
The proportion of conspecific and heterospecific competitors survived with the presence and absence of maternal care of *Cheyletus eruditus*. Different lowercase letters indicate significant difference among treatments at the 5% level.

**Figure 7:**
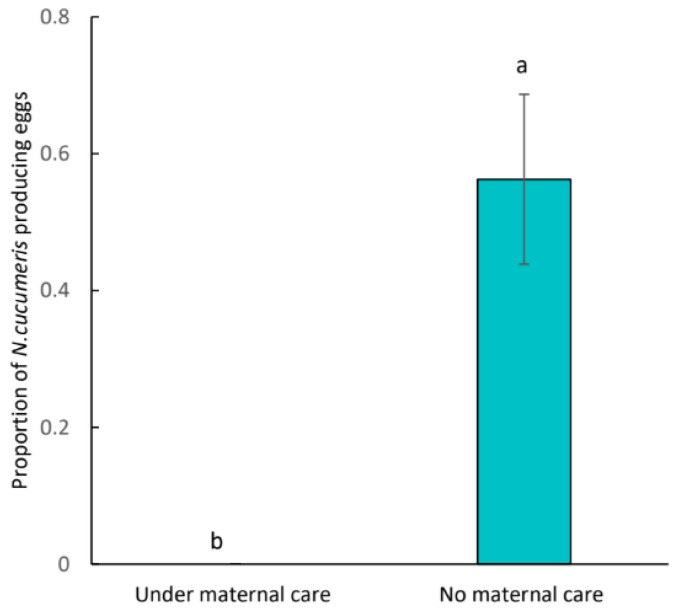
The proportion of the number of cells in which the eggs of heterospecific competitors *Neoseiulus cucumeris* were observed during the experiment. Zero for that under maternal care indicates treatments with no eggs were observed. Different lowercase letters indicate significant difference among treatments at the 5% level.

There was no difference in survivorship of conspecific competitors and heterospecific competitors (*Z* = 0.000; *p* = 1.000). When no *C. eruditus* was present, all the conspecific and heterospecific competitors survived (*p* > 0.05; Figure 6). While under maternal care, death of both conspecific and heterospecific competitors was observed, and the mortality rates did not differ (*p* > 0.05). Across these four treatments, the survival rate of competitors in cells without guarding females was significantly higher than those with maternal care (*p* < 0.001; Figure 6). Additionally, in the presence of *C. eruditus*, no *N. cucumeris* egg was observed. Whereas, about 56% of experimental cells without the presence of *C. eruditus* were found with *N. cucumeris* eggs, and the difference in the proportion of the number of cells with *N. cucumeris* was significant. (χ^2^ = 9.8937, df = 1, *p* = 0.002; Figure 7). 

## 4. Discussion

Understanding life history strategies, such as parental care behavior, is crucial for predicting population dynamics and evaluating the compatibility of combined releases in biological control among agents that exhibit cannibalism and intraguild predation. In this study, the plasticity of maternal caring behavior and its influence on egg survival, as well as on the potential con- and hetero-specific competitors, were explored to fully understand the ecological consequences of egg-guarding behavior in the predatory mite *C. eruditus.* It was found that the majority of gravid females showed egg-guarding behavior, regardless of the presence of potential competitors. In comparison, when a heterospecific competitor was introduced, the mothers were more likely to guard their eggs. The egg mass experienced a significantly higher risk of being attacked without their mother, and maternal care notably reduced their risk of being attacked by con- and heterospecific predators. Maternal care also influenced the survival of potential competitors. Specifically, significantly higher mortality of both con- and hetero-specific predators was observed in the presence of maternal care.

### 4.1. Egg Guarding Behavior and Response to Environment 

Females of *C. eruditus* typically laid eggs in a cluster and guarded them, and only a few females were found away from their egg masses even though they were caged separately without any competitor in the experiment cells. This finding indicated that this behavior has evolved through selection and is highly conserved. It has also been reported that these females guarded the egg masses for a long time [26], suggesting they strive to protect the immobile and most vulnerable stage of their offspring from predation risk. The predominant risk is thought to be cannibalism. This assumption is supported by earlier studies documented that cannibalism is common when the population of the predatory mite is high density. Additionally, when their prey was scarce, the predator population decreased under cannibalism, and only the adult females survived [25]. However, we cannot exclude the possibility that this parental care strategy evolved as a response to intraguild predation or other forms of predation despite the limited information on intraguild predation between *C. eruditus* and other species. This is supported by observations in this study where *N. cucumeris* was seen attacking *C. eruditus* eggs, and the guarding females responded by killing *N. cucumeris*.

While most mothers in this study stayed closely with their egg masses, a few did not, probably engaging in searching for prey. Mothers always need to balance competing demands, notably, the need for food to sustain themselves and the imperative of caring for their eggs to ensure successful reproduction [6,13]. They must balance these demands in response to a range of environmental factors, including social environment and food availability. In this study, we observed that the females altered their behavior in response to the presence of the competitors. However, there is also the possibility that they might have been more vigilant when a heterospecific competitor was present. This assumption was supported by the results that all females guarded their eggs in treatment with *N. cucumeris*. This finding firstly indicated that females of *C. eruditus* are capable of discriminating the cues from heterospecific predators. These unfamiliar cues probably appeared more dangerous, promoting the females to exert more effort into egg guarding. It proved that parenting behavior is greatly influenced by the social environment [6]. It is also possible that the heterospecific competitor might have attacked or simply inspected the guarding mother and the egg mass more frequently. Food availability also influences the mother’s behavior, as evidenced by this species eating their eggs when prey becomes scarce [30]. This phenomenon, also reported in fish, features a low survival rate of eggs when only the caregiving male is present [13], and this phenomenon has been explained as a strategy for enhancing survival and gaining future mating success [36]. 

### 4.2. Ecological Consequence of Egg Guarding 

Egg masses under maternal care are safeguarded from competitor attacks, aligning with our predictions. This result was consistent with a study on the maritime earwig *Anisolabis maritima* (Bonelli). In laboratory experiments, the introduced alien individual of the same species significantly decreases the chances of eggs successfully hatching. However, maternal nest defense has reduced this risk [4]. Although in short-term behavioral studies, maternal guarding behavior increases reproductive success, these benefits may potentially come at the cost of future reproductive success. On the one hand, the guarding behavior may decrease the mating opportunities of sexually reproductive animals. But this assumption does not apply to *C. eruditus*, which reproduces asexually [22]. On the other hand, the guarding females have limited time and space for foraging, which could negatively influence their survival and future reproductive output, which requires further exploration. 

The presence of guarding females negatively influenced the competitors by killing them, including both con- and heterospecific predators, suggesting that to ensure the safety of their offspring, the guarding females attacked and killed the potential competitors. The heterospecific predator showed a 19.3% lower survival rate than the conspecific competitor, although the difference was not yet statistically significant. The difference may result from the divergence in hunting mode. *Neoseiulus cucumeris* usually actively searches for prey [37], while *C. eruditus* was recorded as a sit-and-wait predator and rarely actively hunting for prey [23], leading to a much higher encounter rate between *N. cucumeris* and the guarding female and a higher death rate of *N. cucumeris*. It could also be explained by the size difference between *C. eruditus* and *N. cucumeris.* The former was about 0.5 mm [38], while the female of *N. cucumeris* was 0.45 mm in body length [39]. The smaller body size of *N. cucumeris* likely makes it easier to be killed by *C. eruditus.* This finding further indicated that egg guarding is time-consuming and energy-consuming for *C. eruditus*. Under the maternal care of *C. eruditus*, *N. cucumeris* eggs were not found; probably, the *N. cucumeris* did not produce eggs, or the laid eggs were consumed by *C. eruditus.* This finding implies that these two species are unsuitable for combined release.

## 5. Conclusions

In conclusion, this study has shed light on the prevalence and advantages of maternal care behaviors in the biological control agent *C. eruditus*. It has revealed that females’ enhanced egg-guarding behavior in the presence of a heterospecific competitor increases their egg survival and affects competitor survival rates, indicating that maternal care is a critical factor regulating the population dynamics of these predatory mites. However, this research only determined the maternal care behavior in the presence of two species of competitors over a very short term. Other factors, such as prey availability and the long-term consequences, have yet to be explored. Furthermore, the trade-off between maternal care and foraging opportunities presents an area that requires further exploration to comprehend its impact on future reproductive success and lifetime fitness. These further investigations have the potential to significantly improve the implementation and effectiveness of biological control programs.

## Data Availability

All relevant data are presented in the paper. Additional data can be supplied upon request.
